# Growth and development of *Arabidopsis thaliana* under single-wavelength red and blue laser light

**DOI:** 10.1038/srep33885

**Published:** 2016-09-23

**Authors:** Amanda Ooi, Aloysius Wong, Tien Khee Ng, Claudius Marondedze, Christoph Gehring, Boon S. Ooi

**Affiliations:** 1 Division of Biological and Environmental Sciences and Engineering, 4700 King Abdullah University of Science and Technology, Thuwal 23955-6900, Kingdom of Saudi Arabia; 2 Division of Computer, Electrical, and Mathematical Sciences and Engineering, 4700 King Abdullah University of Science and Technology, Thuwal 23955-6900, Kingdom of Saudi Arabia; 3 Cambridge Centre for Proteomics, Cambridge Systems Biology Centre, and Department of Biochemistry, University of Cambridge, Cambridge, CB2 1GA, United Kingdom

## Abstract

Indoor horticulture offers a sensible solution for sustainable food production and is becoming increasingly widespread. However, it incurs high energy and cost due to the use of artificial lighting such as high-pressure sodium lamps, fluorescent light or increasingly, the light-emitting diodes (LEDs). The energy efficiency and light quality of currently available horticultural lighting is suboptimal, and therefore less than ideal for sustainable and cost-effective large-scale plant production. Here, we demonstrate the use of high-powered single-wavelength lasers for indoor horticulture. They are highly energy-efficient and can be remotely guided to the site of plant growth, thus reducing on-site heat accumulation. Furthermore, laser beams can be tailored to match the absorption profiles of different plant species. We have developed a prototype laser growth chamber and demonstrate that plants grown under laser illumination can complete a full growth cycle from seed to seed with phenotypes resembling those of plants grown under LEDs reported previously. Importantly, the plants have lower expression of proteins diagnostic for light and radiation stress. The phenotypical, biochemical and proteome data show that the single-wavelength laser light is suitable for plant growth and therefore, potentially able to unlock the advantages of this next generation lighting technology for highly energy-efficient horticulture.

Indoor horticulture can contribute to solutions for sustainable food production and is particularly appealing not only to ‘horticultural unfriendly’ regions that experience water scarcity, have limited areas of arable land (e.g. due to climate change or urban development), or receive insufficient amounts of natural sunlight due to their geographical locations^1^,^2^. Indoor farming enables plant production to be carried out all-year-round in a highly controlled growth environment that requires minimal water consumption and space especially when conducted in space-saving multi-tiered vertical growth settings that is particularly attractive in dense urban areas. Thus, indoor farming allows crops to be cultivated at any time irrespective of the weather patterns to meet the demands of a growing world population^3^,^4^. Commercial indoor farming relies heavily on artificial lighting employing conventional broad-spectrum sources such as the high-pressure sodium (HPS) and metal halide (MH) lamps, fluorescent lights and increasingly, the narrow spectrum light-emitting diodes (LEDs). The currently used light sources are inefficient because of their low light-to-heat output and the suboptimal light qualities for plant growth. Current lightings also incur high energy cost that may include the cost for extensive cooling to offset the high heat radiant output and this makes these lightings unsuitable for cost-effective large-scale plant production^5^.

Recent advancement in solid-state lighting (SSL) technologies has resulted in a significant contribution to the development of horticultural illuminants, such as LEDs for indoor plant cultivation in highly controlled environments and for space-based plant growth systems in NASA’s Advanced Exploration Systems Habitation Projects (for review see^6^,^7^,^8^). Application of LED lighting in plant growth was first documented in lettuce (*Lactuca sativa* L. cv. Grand Rapids)^9^, in which the growth and development under monochromatic red LED (660 nm) supplemented with blue fluorescent lamps (400–500 nm) was comparable to that under cool-white fluorescent and incandescent lights. Thereafter and concomitant with the advancement of LED technology, there has been a surge in interest in the application of LED lighting in horticulture (for review see 2,7,8,10,11). Red and blue lights are the photosynthetically dominant wavelengths that are efficiently absorbed by plants to promote their growth and development^10^,^12^. The combination of both red and blue monochromatic LEDs at different wavebands and light intensities for the growth and development of green vegetables such as lettuce (*Lactuca sativa*)^9^,^13^,^14^,^15^,^16^, spinach (*Spinacia oleracea*)^17^, cabbage (*Brassica oleracea*)^18^,^19^ and cucumber (*Cucumis sativus*)^20^,^21^ as well as herbal plants^22^ has been reported previously. Despite the many advantages of LED application for indoor plant cultivation, the electrical-to-optical power conversion of LEDs remains inefficient beyond a certain electrical current.

Semiconductor lasers on the other hand, promise unparalleled advantages over existing illumination technologies^23^,^24^ (Fig. 1a). Firstly, the input power density-to-optical light output of laser is higher than the current horticultural lighting because laser diodes (LDs) have much greater power conversion efficiency (PCE) than for example the LEDs especially at high current densities of ≥10 kWcm^−2^ 25,26. While the blue and red LEDs have PCEs of up to 60 and 40% respectively^27^, they incur a drastic loss of PCE at input power density of ≥1 kWcm^−2^ 26. For instance, with increasing input power densities from 4 to 10 kWcm^−2^, the PCE of the blue LD remains close to 30% whilst a significant drop in efficiency from 20 to 10% is observed for blue LED^25^,^26^. This ‘efficiency droop’^25^,^28^ renders LEDs inefficient for large-scale high intensity lighting applications e.g. in horticulture. Since laser luminaires have a long lifespan and are suitable for directional emissions and operation at higher current densities, a higher photon flux density can be achieved. This in turn translates into the manufacturing of more cost- and space-efficient illumination devices that afford the use of smaller electronic chips^26^,^29^. Laser diodes on the other hand are small size, durable and are able to operate at higher optical output power with ease of operation and manipulation and at comparable costs as compared to LEDs^29^. Recently, the use of projector laser scanner consisting of laser diodes (50 to 100 mW) combined at three wavelengths (450 nm, 570 nm and 640 nm) in comparison to fluorescent lamp has been shown to be sufficient to grow radish sprouts by directing the emitted photons to the leaf surface^30^. Fluorescent lighting is less efficient as compared to laser as it emits several discrete wavelengths ranging from 350 nm to 750 nm in all directions and many of these wavelengths do not match the absorption profile of the plant photosynthetic apparatus^5^. Therefore, laser technology promises increased energy-efficiency and potentially cost-saving alternative artificial light source for small spaces (e.g. space-base plant growth and human life-support) and industrial-scale horticultural applications^29^,^30^,^31^. Secondly, unlike the conventional light sources, the narrow beam angle of laser light enables illumination over far distances thus allowing light to be generated remotely, eliminating the need to mount light panels directly above the plant growth area. Since the laser light source can be externally placed, indoor laser-based horticulture can reduce undesirable on-site heat accumulation that is commonly associated with the currently used artificial lighting (Fig. 1a). This cool-emitting feature of laser light is economically attractive especially in larger enclosed growth spaces where extensive cooling that consumes both energy and water, is employed^5^,^32^. These attributes and the high-power capability of lasers offer the prospect of cost- and space-savings especially in a vertical horticulture setting where multiple-tiered growth spaces can be illuminated by a single laser light source that is guided through optical fibres or free space from a remotely installed parent laser illumination system (Fig. 1c)^24^. Thirdly, laser beams are highly tunable^31^ where the wavelength and intensity of individual single waveband laser can be customized to specifically match the absorption profiles of different plant species and growth phases to enhance economically relevant traits^8^,^33^,^34^. This high degree of flexibility can give rise to new lighting architectures as laser beams can be focused, steered and mixed for optimal results^24^.

Red laser diode with a peak emission at 680 nm with 500 mW output power supplemented with 5% of blue fluorescent light has been previously applied to grow rice (*Oryza sativa* L. cv. Kitaibuki) from the early vegetative to the seed yield stage. However, this light regime resulted in both lower tiller spikes and seed yield^31^. Furthermore, another red laser diode at 650 nm with 7 mW output was used as a supplementary lighting to grow egg plant and sweet pepper^29^. In this case, the authors do not state the nature of any additional light sources and it is not conceivable that the reported growth parameters could have been achieved in the absence of blue light^29^. In contrast to the previous reports that utilise laser as supplementary lighting for plant cultivation, we demonstrate the use of single-wavelength laser light as exclusive light source for indoor plant cultivation by applying our laser illumination system on the growth and development of *Arabidopsis thaliana* (ecotype Col-0) model plant. We show that these plants are able to complete a full growth cycle from seed to seed solely under single waveband red and blue lasers.

## Results and Discussion

### Design and assembly of a laser-illuminated plant growth chamber

**Figure 1:**
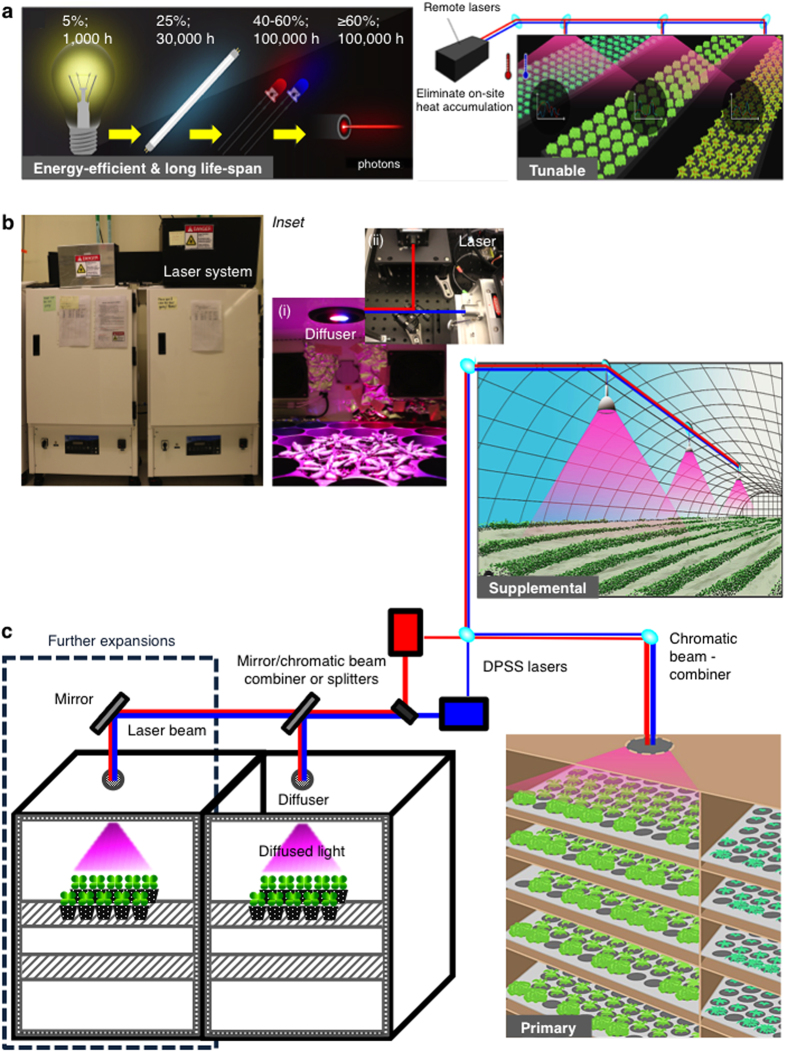
A laser-illuminated plant growth chamber prototype and its beneficial attributes for horticultural applications. (**a**) Beneficial attributes of single-wavelength laser light for horticulture^27^,^64^. (**b**) A laser-illuminated plant growth chamber prototype used in this study. *Inset*: (i) Light distribution (magenta in color) of the laser illuminated growth area upon passing through the engineered diffuser. (ii) Position of the red (671 nm) and blue (473 nm) DPSS lasers and the optics inside the protective black metal case. (**c**) Schematic illustration of the prototype and the potential applications of laser as primary and supplementary lighting for horticulture and light-related research. The laser modules and optics are installed external of a custom-made growth chamber (Percival Scientific, Perry, IA) and are enclosed in a protective black metal case. The laser illumination system consists of two diode-pumped solid-state (DPSS) lasers (maximum power output: >500 mW; Class IV; Laserglow technologies, Toronto, Canada) that generate a 9:1 ratio of red (671 nm) and blue (473 nm) laser beams that are combined at a 1.27 cm short-pass dichroic mirror (with a cut-off wavelength at 589 nm) and guided a 1-inch diameter multiple-ground glass engineered diffuser with a 50-degree divergence angle that is custom-fitted at an opening on the roof of the chamber providing a non-Gaussian magenta-colored square light-pattern distribution illuminating an area of 227 cm^2^ that is fixed at 20 cm vertically below the diffuser.

We describe the design, assembly and installation of a laser illumination system in a prototype plant growth chamber. This laser illumination system consists of two diode-pumped solid-state (DPSS) lasers (Laserglow Technologies, Toronto, Canada) that generate single-wavelength laser beams, adjusted to a ratio of 9:1 of red (671 nm) : blue (473 nm), giving an average of total photon flux density of 90–100 μmol m^−2^ s^−1^ (see Methods). These two laser beams emit the photosynthetic dominant wavelengths that correspond to the absorption peaks of light-harvesting antennal pigments^1^,^6^,^35^. To investigate the effect of light quantity and quality such as the PPFD (μmol m^−2^ s^−1^), wavelengths (nm) and ratio of light compositions that are optimal for growth and development of *Arabidopsis thaliana*, we first assayed seedling emergence by measuring the hypocotyl length under different regimes of LEDs (Supplementary Fig. S1). We confirmed that the composition of red to blue LEDs at 9:1 ratio (Supplementary Fig. S2) is best suited for the growth of *Arabidopsis*. This is consistent with previous studies that used the same light ratio to grow lettuce^9^,^14^,^17^, spinach^17^ and cucumber^21^, and we therefore used these light parameters for our laser illumination prototype. It has been reported^16^,^21^ that while increasing blue light fraction promotes photosynthetic efficiency for optimal plant productivity, only 7% of blue light was sufficient to prevent dysfunctional photosynthesis^20^. In this system (Fig. 1b), both the red and blue laser beams are combined when the beams converged at a 1.27 cm short-pass dichroic mirror (with a cut-off wavelength at 589 nm), guided through free space to a reflector secured above an opening on the roof of the chamber approximately 30 cm away from the first dichroic mirror. The reflector directs the combined laser beams perpendicularly downwards through an opening at the roof of the custom-built plant growth chamber (Percival Scientific, Perry, IA) that is tightly fitted with a 1-inch diameter multiple-ground glass engineered diffuser with a 50-degree divergence angle (ED1-S50, 90% transmission spectrum from 380 to 1100 nm wavelength) (Thorlabs Inc., Newton, NJ). Upon passing through the diffuser, the emitted red and blue laser beams diffuse and produce a non-Gaussian magenta-colored square light-pattern distribution illuminating an area of 227 cm^2^ that is fixed at 20 cm vertically below the diffuser (Fig. 1c). Homogeneity of the light intensity distribution is largely dependent on the characteristics of the diffuser and in this case is limited by the size and the nature of diffuser. Consequently, the light intensities decrease with increasing distance from the central position of the diffuser^29^. We took an average of light intensities measured at five different horizontal points within the illuminated area, adjusted accordingly to the fixed total PPFD of 90–100 μmol m^−2^ s^−1^ to account for the intensity differences (see Methods). The spectral characteristics such as the dominant wavelengths, the ratios and color spaces of both the white fluorescent and laser light used in this study are given as spectral composition and chromaticity diagrams (Supplementary Fig. S3). We tested this laser illumination prototype on the *Arabidopsis thaliana* model plant and noted that the plants appeared to be healthy and were able to complete a full growth cycle from seed germination (Supplementary Fig. S4) to the production of viable seeds (Supplementary Fig. S5).

### Transcriptional responses of photosynthetic and light stress marker genes

**Figure 2:**
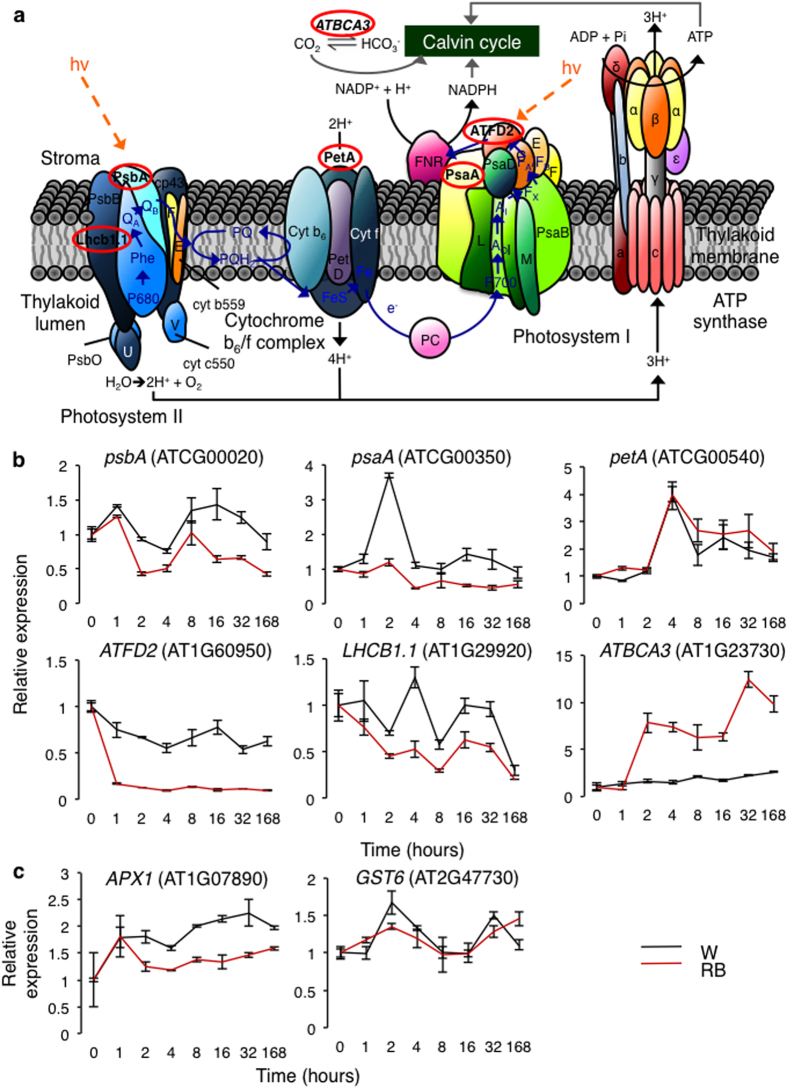
Expression of marker genes implicated in photosynthesis and light stress. (**a**) Plant photosynthetic pathway. (**b**) Six photosynthetic marker genes, *photosystem II reaction center protein A, psbA* (ATCG00020.1), *photosystem I P700 chlorophyll A apoprotein A1, psaA* (ATCG00350.1), *photosynthetic electron transfer A, petA* (ATCG00540.1), *ferredoxin 2, ATFD2* (AT1G60950.1), *chlorophyll A/B binding protein 1.1, LHCB1.1* (AT1G29920.1) and *beta carbonic anhydrase 3, ATBCA3* (AT1G23730.1), each representing the main components of the photosynthetic pathway, are selected for expression study using semi-quantitative PCR. (**c**) Expression levels of two genes implicated in light stress, *L-ascorbate peroxidase 1, APX1* (AT1G07890.3) and *glutathione S-transferase phi8, GST6* (AT2G47730.1). 21-days old *Arabidopsis* plantlets grown under 90–100 μmol m^−2^ s^−1^ of cool-white fluorescent (W) light for 16/8-hour photoperiod at 22 °C with a relative humidity of 50–60% were illuminated with a continuous regime of red and blue (RB) laser light for seven days. Rosette leaves from three different biological replicates were harvested at 0, 1, 2, 4, 8, 16, 32, and 168 hours, after which the RNA were isolated and cDNA synthesized for the gene expression studies. All data were normalized against the *protein phosphatase 2A subunit A3, PP2AA3* (AT1G13320) gene. Error bars represent standard error of the mean calculated from three independent biological replicates.

We examined a number of molecular parameters in plants grown under single-wavelength laser light including the expression levels of six photosynthetic marker genes, each representing a main component of the photosynthetic pathway (Fig. 2a,b). With the exception of *beta carbonic anhydrase 3 (ATBCA3*) and *photosynthetic electron transfer A (PetA*), the laser-grown plants have a consistently lower expression of photosynthetic genes across all time points as compared to the control plants grown under cool-white fluorescent light (Fig. 2b). Since the laser light provides wavelengths that closely match the main absorption peaks of the photosynthetic pigments, a more efficient productivity can be achieved as thermal dissipation of light absorbed in the photosynthetically inefficient wavebands is avoided^7^. This could account for the lower expression of the photosynthetic marker genes including the *light harvesting chlorophyll A/B-binding protein 1.1 (LHCB1*) (Fig. 2b) as well as the reduced chlorophyll content in laser-grown plants. Expression of chloroplast *photosystem II reaction center protein A (psbA*) gene is generally associated with photo-inhibition and the photo-damaged of photosystem II (PSII)^36^,^37^,^38^ especially when the light absorption exceeds consumption^36^,^37^. Notably, the laser-grown plants have lower expression of *psbA* (Fig. 2b), which suggests reduced photo-inhibition under this light regime. The expression of two light stress marker genes, *ascorbate peroxidase 1 (APX1*) and *glutathione s-transferase (GST6)*^36^ was lower in the plants grown under laser light regime (Fig. 2c) implying that the laser illumination conditions induce less stress than the white fluorescent light at similar PPFD. This is also in agreement with the reduced expression of *psbA*, a gene that is diagnostic for photo-inhibition^38^. Importantly, anthocyanin accumulation associated with damaging radiations^38^,^39^,^40^ on the leaf and stem of the laser-grown plants is reduced, indicating that the laser light regime is not only suitable for photosynthesis and photo-morphogenesis, but also causes less stress than the white fluorescent light. We also noted a high expression of *ATBCA3* in the laser-grown plants (Fig. 2b). ATBCA3 is involved in carbon utilization during photosynthesis, generating carbon dioxide (CO_2_) from bicarbonate to provide optimal CO_2_ level at the carboxylation site of ribulose 1,5-bisphosphate carboxylase/oxygenase (Rubisco) by facilitating CO_2_ diffusion across the chloroplast envelope or through the rapid dehydration of bicarbonate to CO_2_^41^,^42^. ATBCA3 also has non-photosynthetic roles such as lipid biosynthesis for seedling survival^43^ and for the control of guard cell aperture^44^,^45^,^46^. Taken together, the gene expression data suggest the suitability of the laser light regime for plants to operate at a high photosynthetic efficiency, presumably synthesizing sufficient NADPH and ATP for CO_2_ fixation and for cellular processes downstream of photosynthesis. In contrast, plants that are grown under high light condition will generate excess NAPDH in the stroma, resulting in the accumulation of potentially harmful excitation energy in the photosynthetic membrane^47^,^48^.

### The proteome of plants grown under laser light

**Figure 3:**
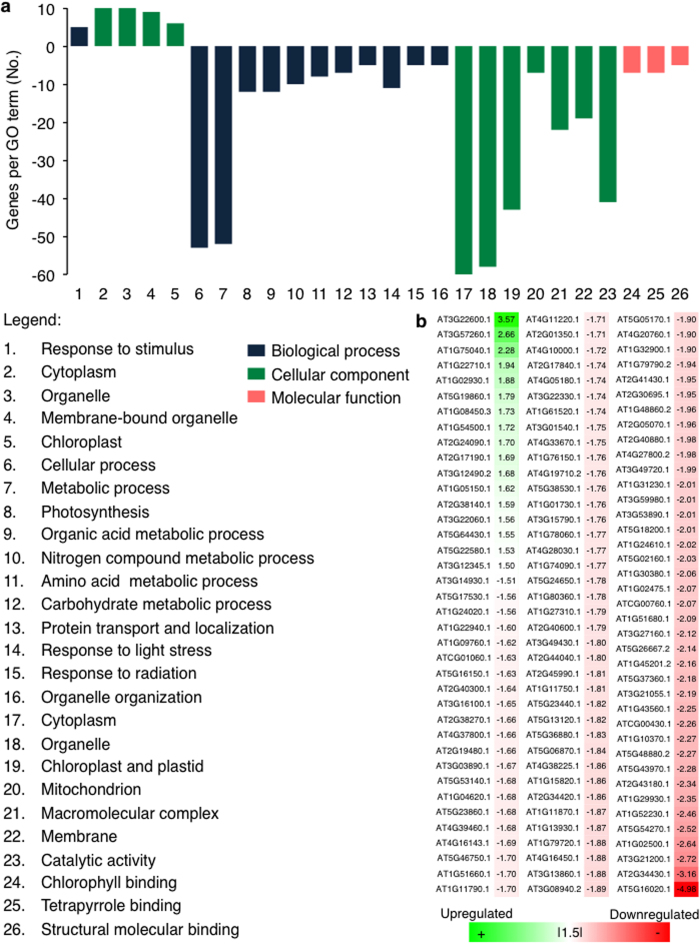
Comparative proteome analysis of laser-grown Arabidopsis plants. (**a**) Functional classification of differentially expressed *Arabidopsis* proteins in response to single-wavelength red and blue lasers light. Total soluble proteins were extracted from *Arabidopsis* plantlets treated under a continuous laser light for 7 days with average photon flux density of 90–100 μmol m^−2^ s^−1^. Protein extraction was done with tricarboxylic acid (TCA) precipitation prior to iTRAQ labeling for liquid chromatography-tandem mass spectrometry (LC-MS/MS). Proteins that have *P* value of ≤ 0.05 and fold change of Ι1.5Ι were considered as differentially expressed (see Methods for data analysis). (**b**) List of differentially expressed proteins in laser-grown plants.

To further examine the influence of single-wavelength red and blue laser light on plant growth at cellular translational level, we have undertaken a comparative analysis of the proteomes of laser- and white light-grown plants to reveal changes at the systems level that might be diagnostic for structural or functional changes induced by the different light regimes. Our study revealed that the laser-grown plants have 115 differentially regulated proteins of which the majority (98) are down-regulated (Fig. 3a). Among the 17 proteins that are up-regulated, there is no significant enrichment of biological processes. However, most of the proteins that are increasing in abundance have roles in metabolic processes (6 proteins) and in response to abiotic stress (5 proteins). Of the down-regulated proteins, 43 are annotated as localized in the ‘chloroplast and plastid’, 12 are involved in ‘photosynthesis’ and 14 are involved in ‘chlorophyll and tetrapyrrole binding’ (Fig. 3a). There is an over-representation of down-regulated proteins that are associated with light radiation. This set of proteins includes seven light-harvesting chlorophyll-protein (LHC) complexes of which LHCB1.4 (AT2G34430) and LHCB3 (AT5G54270) are among the most down-regulated proteins in laser-illuminated plants. These proteins are a component of the main light harvesting chlorophyll a/b-protein complex of PSII that function as light receptors. They are involved in fine-tuning the amount of light energy to be channeled to the reaction centers, enabling plants to adapt to a wide-spectrum of light environments to drive the processes of photosynthesis^48^. Similar to previous findings^49^,^50^,^51^, LHC family proteins were observed to be light-stress induced and consequently, a decrease in the abundance of photosynthetic machinery-associated proteins is indicative of reduced photo-oxidative stress or light induced stress under laser-illuminated plants. This finding is consistent with the expression pattern of the examined photosynthetic marker genes, particularly the *LHCB1* gene under the laser light regime (Fig. 2b). Importantly, 16 proteins that have a role in the ‘response to light stress or radiation’ are down-regulated in the laser-illuminated plants. Thus, the reduced expression of the corresponding light stress marker genes, *APX1* and *GST6* examined in this study (Fig. 2c) lends support to the notion that the laser light regime induces less stress than white fluorescent light. In addition, the proton gradient regulation 7 protein (PGR7, AT3G21200) is also down-regulated. *Arabidopsis thaliana* PGR7 has been shown to be involved in both PSII and P700 of photosystem I and is necessary for efficient photosynthetic electron transport^52^. Photosynthetic electron transport plays functional roles in light-dependent NAPDH and ATP synthesis as well as in photoprotection during high light condition by generating a high pH gradient across the thylakoid membrane for thermal dissipation of excess light energy. Compared to the wild type, the rate of electron transport between PSII and PSI was reduced with a significant change in the redox state of the first stable electron acceptor of PSII (Q_A_) in *Arabidopsis pgr7* mutant without affecting the cytochrome *f* accumulation. The P700 was more oxidized in the *pgr7* mutant at light intensities > 150 μmol m^−2^ s^−1^ indicating that PGR7 can modulate electron transport between Q_A_ of PSII and P700 of PSI^52^. Furthermore, the chlorophyll content measured in *pgr7* mutant is lower^52^, suggesting that the down-regulation of this protein could explain the lower expression of the photosynthetic marker genes and the reduced chlorophyll level in laser-illuminated plants. Taken together, the expression patterns of the examined molecular marker genes and the absence of elevation of light-stress response proteins in the proteomics data (Fig. 3b) lend further support to the suitability of the current laser light regime for plant growth.

### Phenotypic and biochemical characterisation of plants grown under laser light

**Figure 4:**
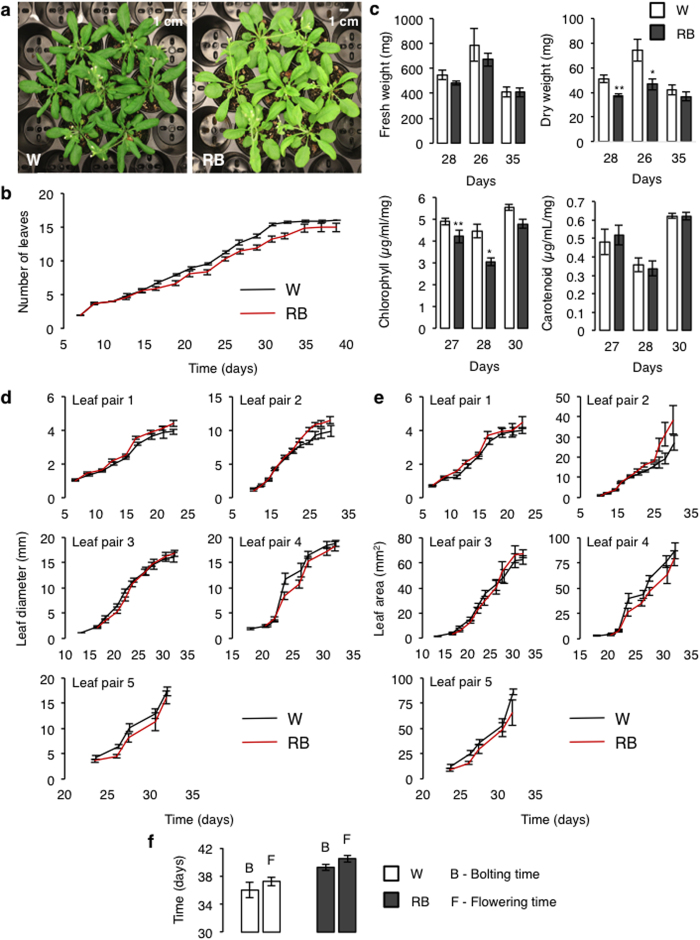
Phenotypic and biochemical characterizations of plants grown under laser light. (**a**) 30-days old *Arabidopsis thaliana* grown under white fluorescent (W) and single-wavelength red and blue (RB) laser light respectively. (**b**) Leaf development (leaf count) of plants that are fully-grown under the laser light regime. (**c**) Measurement of fresh and dry weights and biochemical analysis (total chlorophyll and carotenoid quantification) of *Arabidopsis* plants exposed to a continuous (RB) laser light for seven days. (**d**) Diameter and (**e**) surface area of rosette leaf pair of plants fully-grown under (RB) laser light. (**f**) Bolting and flowering time of plants grown under laser. All data collected with the exception for (**c**), were obtained from plants that were grown from the first day of sowing to the completion of growth cycle under (RB) laser light only (9:1 ratio of red (671 nm) and blue (473 nm) lasers) at an average photon flux density of 90–100 μmol m^−2^ s^−1^ for 16/8-hour photoperiod at 22 °C with a relative humidity of 50–60%. For (**c**), *Arabidopsis* plants were germinated and grown under cool-white fluorescent light at similar light intensity and growth condition for three to four weeks prior to exposure to a continuous (RB) laser regime for seven days. Both the leaf diameter and surface area were analyzed and measured using ImageJ software^58^. Error bars represent standard error of the mean calculated from *n* > 10 for (**b, d** and **e**), where *n* represents the number of leaves from seven independent plant replicates (*n* = 7 (**c**) and *n* = 3 (**f**)) where *n* represents the number of independent biological replicates. One asterisk (*) signifies *P* < 0.05 and two asterisks (**) signify *P* < 0.005.

Plants grown under this specific laser light regime show phenotypic traits that differ only slightly from those grown under white fluorescent light (control) under similar growth conditions (Fig. 4a) and are in fact much like those reported for plants grown under similar monochromatic light with a broader spectra provided by the LEDs^5^,^32^ (Supplementary Figs S1 and S2). In laser-grown plants, the emergence of new leaves is delayed (Fig. 4b) and the leaves have lower total chlorophyll but not carotenoid content (Fig. 4c) consistent with a lighter shade of green observed. They also have a reduced dry weight while their fresh weight does not differ significantly from that of white light grown plants (Fig. 4c). It was reported^17^ that chlorophyll levels and shoot dry weight in spinach (*Spinacea oleracea* L. cv. Nordic IV) are reduced under a 9:1 ratio of red LED (660 nm) supplemented with blue fluorescent lamp at total PPFD of 282 μmol m^−2^ s^−1^ as compared to plants grown under cool-white fluorescent light. Furthermore, the proportion of blue light fraction added to the light condition can affect chlorophyll content. It has been shown that the chlorophyll content increases with decreasing red:blue light ratio^16^,^21^. The average leaf length and area of the first two leaf pairs are higher than the control plants and equal or lower in the subsequent leaf pairs (Fig. 4d–e) while bolting and flowering times are also slightly delayed (Fig. 4f). The delay in flowering time is most likely a consequence of the absence of far-red light that promotes flowering^53^,^54^. The absence of photosynthetically inefficient far-red as well as green light is likely to exhibit some effects on the growth and development since both these wavelengths can also oppose the growth-promoting properties of red and blue light in vegetative development, photoperiodic flowering, stomatal opening and stem growth modulation^35^,^55^. Plants do not require the wavebands of the entire light spectrum for their growth and development, in fact, they have evolved optimized light capture mechanism to grow under a given specific set of light conditions^7^,^56^,^57^. Given these considerations, further experimentation with additional wavelengths supplemented at specific times of the day and/or developmental phase will have to be tested in the future before the high power and energy-efficient attributes of lasers can be fully harnessed for horticultural applications.

In conclusion, we have developed a prototype laser-illuminated growth chamber and showed that the application of diffused single-wavelength red and blue laser light is sufficient for plant growth and development. This will provide a basis for further optimization of laser light technologies for optimal plant growth. Given the potential benefits of laser light^23^,^24^, we foresee that this technology will eventually be used in plant factories and drive highly energy-efficient plant cultivation.

## Methods

### Design, assembly and installation of a laser illumination system

Pre-aligned laser beams emitted from the red and blue DPSS laser source respectively (maximum power output: >500 mW; Class IV; Laserglow technologies, Toronto, Canada) were combined using a 1.27 cm diameter short-pass dichroic mirror (cut-off wavelength at 589 nm) attached to an adjustable kinematic mount (Edmund Optics, Barrington, NJ) that is remotely placed outside of the custom-built plant growth chamber (Percival Scientific, Perry, IA). This mirror allows the shorter wavelength blue laser beam to pass through but reflects the longer wavelength red laser beam at 90° to achieve parallel beam paths that converge at a reflector mirror mounted externally above an upper opening at the roof of the chamber. Both collimated (non-dispersive) red and blue laser beams are then reflected 90° downward onto a 1-inch diameter multiple-ground glass engineered diffuser with a 50-degree divergence angle (ED1-S50, 90% transmission spectrum from 380 to 1100 nm wavelength) (Thorlabs Inc., Newton, NJ) that was custom-fitted at the bottom opening on the roof of the chamber. Upon passing through the diffuser, the emitted red and blue laser beams diffuse and produce a non-Gaussian magenta-colored square light-pattern distribution illuminating an area of 227 cm^2^ that is fixed at 20 cm vertically below the diffuser. The distribution of light intensity covering the illuminated area was determined by taking an average of light intensities at five different horizontal points within the illuminated area fixed at 20 cm vertically below the diffuser. Here, we adjust the light intensity to an average total photon flux density of 90–100 μmol m^−2^ s^−1^ consisting of 9:1 ratio of red (671 nm) and blue (473 nm) laser light. Due to the limitation imposed by the type of diffuser used and the small size of our growth chamber prototype, only seven *Arabidopsis* plants can be grown at the same time in our prototype at the present. The light intensity (Photosynthetically Active Radiation (PAR) in μmol of photons m^−2^ s^−1^) was measured with a LI-250A light meter (LI-COR^®^, Lincoln, NE) equipped with the LI-190R quantum sensor. The irradiance spectra and the corresponding chromaticity diagrams of the laser and cool-white fluorescent light were measured *in situ* using a GL SPECTICS 5.0 Touch spectrometer (JUST Normlicht GmbH, Weilheim an der Teck, Germany) within the waveband range from 340 to 850 nm. The graphs were plotted using a OriginPro 8.5.1 SR2 software (OriginLab Corporation, Northampton, MA). We have also created a program using the Labview software to achieve automated and continuous control of a pre-defined light regime.

### Plant material and growth conditions

*Arabidopsis thaliana*, ecotype Columbia (Col-0) seeds were stratified at 4 °C in the dark for at least four days prior to sowing and growing on a growth medium consisting of a mixture of Jiffy plant starter pellets (Jiffy Products (NB) Ltd., New Brunswick, Canada) and vermiculite at a ratio of 3:1 in 5.08 cm pots. Plants were regularly watered at intervals of three to four days or as needed depending on the plant developmental stage. The temperature and relative humidity of the growth environment were maintained at 22 °C and at 50–60% respectively. For a complete growth cycle under the laser light regime, stratified *Arabidopsis* Col-0 seeds were germinated and grown in our chamber prototype for a 16/8-hour photoperiod light cycle under an average total photon flux density of 90–100 μmol m^−2^ s^−1^, consisting of a ratio of 9:1 of red (671 nm): blue (473 nm) single-wavelength laser light. Plants that were germinated and grown under cool-white fluorescent light at the similar light intensity were used as controls. *Arabidopsis* plants that were used for the measurement of fresh and dry weights, biochemical content, gene expression and comparative proteomics studies were treated under a continuous regime of laser light for a period of seven days in the chamber prototype under an average radiant flux of 90–100 μmol m^−2^ s^−1^. These plants were initially germinated and grown under 16/8-hour photoperiod of cool-white fluorescent light at similar light intensity for three to four weeks prior to the laser light regime.

### Physical and biochemical measurements

#### Soil-based phenotypic analysis

Visual observation or inspection was performed every two days beginning from seed germination and continuously throughout the plant growth and development specifically noting the leaf morphology, leaf number and area and bolting and flowering times.

#### Shoot fresh and dry weights

Shoots of *Arabidopsis thaliana* (ecotype Col-0) were harvested and the fresh weight was determined after seven days of laser light illumination. The plant material was then dried in at 65 °C until a consistent dry weight is obtained.

#### Leaf diameter and surface area

Images of *Arabidopsis* Col-0 plantlets fully-grown in the laser chamber prototype were photographed daily and the leaf diameter and surface area were measured using ImageJ software^58^.

#### Total chlorophyll and carotenoid contents

Rosette leafs of a similar size were harvested from different plants exposed to white light or continuous laser light for seven days. The total chlorophyll and carotenoid content were determined as described previously^59^.

### Gene expression study

#### RNA extraction and cDNA synthesis

RNA from rosette leaves of 21-days old Col-0 were harvested and extracted at 0, 2, 4, 8, 16, 32 and 168 hours upon illumination under single-wavelength red and blue laser light using the RNeasy Mini kit (Qiagen, Germantown, MD) according to the manufacturer’s instructions. The total RNA extracted was quantified and the quality assessed using NanoDrop 2000 UV-Vis spectrophotometer (Thermo Fisher Scientific, Marietta, OH). cDNA was synthesized from 2.5 μg of the extracted RNA using Superscript First-Strand Synthesis and Oligo (dT) for RT-PCR (Life Technologies, Carlsbad, CA) followed by semi-quantitative RT-PCR with gene specific primers (Table 1).

#### Determination of photosynthetic and light stress-related gene expression by semi-quantitative RT-PCR

The expression of photosynthetic and stress-related genes in the laser-grown Col-0 plants was measured by semi-quantitative RT-PCR using the KAPA Taq PCR kit (KAPA Biosystems, Wilmington, MA) on Veriti^®^ 96-Well Thermal Cycler (Applied Biosystems^TM^, Thermo Fisher Scientific, Carlsbad, CA), gene specific primers (Table 1) and PCR cycles according to the manufacturer’s instruction. The photosynthesis-related genes studied were *photosystem II reaction center protein A, psbA* (ATCG00020.1), *photosystem I P700 chlorophyll A apoprotein A1, psaA* (ATCG00350.1), *photosynthetic electron transfer A, petA* (ATCG00540.1), *ferredoxin 2, ATFD2* (AT1G60950.1), *chlorophyll A/B binding protein 1.1, LHCB1.1* (AT1G29920.1) and *beta carbonic anhydrase 3, ATBCA3* (AT1G23730.1). In this study, two general plant stress genes that are indicative of light stress, *L-ascorbate peroxidase 1, APX1* (AT1G07890.3) and *glutathione S-transferase phi8, GST6* (AT2G47730.1) were selected as molecular markers for stress diagnosis. The ImageLab software (Bio-Rad Laboratories, Hercules, CA) was used to quantify the expression of the genes by normalizing against that of protein phosphatase 2A subunit A3, *PP2AA3* (AT1G13320) ‘housekeeping’ gene.

### Comparative proteome analysis

#### Total protein extraction

Leaves were harvested and weighed (approximately of 0.5–1.0 g) prior to total soluble protein extraction with 10% (w/v) tricarboxylic acid (TCA) in acetone. The leaves were re-suspended in TCA solution and lysed by mechanical grinding of two times on ice and incubated at −20 °C overnight. The remaining TCA extraction solution were removed by centrifugation at 3901 × *g* at 4 °C for 20 min. The pellet containing the extracted proteins were washed three to four times with 80% (v/v) acetone sequentially by centrifugation at 3901 × *g* at 4 °C for 20 min to remove remaining TCA solution, unbroken cells and debris. The pellet was then air dried at room temperature (RT) for 15 min prior dissolving in urea lysis buffer (7 M urea, 2 M thiourea, pH 8.0) in 1:3 ratio at RT with shaking for 2–4 hrs. The protein amount was estimated by Bradford method^60^.

#### Protein digestion and iTRAQ labeling

Approximately 100 μg of proteins from both the laser- and white-fluorescent (control) grown *Arabidopsis* plantlets were reduced with 5 mM DL-dithiothreitol (DTT) for 2 hrs at 37 °C, and alkylated with 14 mM iodoacetamide (IAA/IOA) for 30 min at RT in the dark. The alkylation reaction was subsequently terminated by adding to a final concentration of 10 mM of DTT to quench the unreacted IAA/IOA at RT, dark condition for 15 min. The samples were then diluted sevenfold with 50 mM triethylammonium bicarbonate (TEAB) prior to digestion with trypsin (Promega, Madison, WI) overnight at 37 °C in a 1:25 trypsin-to-protein mass-ratio. The protein digests were desalted using Sep-Pak C18 cartridges (Waters, Milford, MA) and dried in a SpeedVac (Thermo Electron, Waltham, MA). The digested protein samples were labeled with iTRAQ reagents according to the manufacturer’s protocol (Applied Biosystems, Framingham, MA). The desalted digests were reconstituted in 30 μL of 1 M iTRAQ dissolution buffer and mixed with 70 μL of ethanol-suspended iTRAQ reagents (one iTRAQ reporter tag per protein sample). The samples were labeled with the respective tags as following: two control represented by two biological replicates for white fluorescent-grown plants were labelled with reporter tags 114 and 116 respectively; and the two laser-grown plants samples with reporter tags 115 and 117. Labeling reactions were carried out at RT for 60 min prior pooling into a single tube and dried in a SpeedVac.

#### Strong cation exchange fractionation of peptide mixture and mass spectrometric analysis using liquid chromatography-tandem mass spectrometry (LC-MS/MS)

All steps were performed according to the methods described previously^61^,^62^.

#### Data analysis

All acquired spectra were submitted to MASCOT search engine (Matrix Science, London, UK) for protein identification as described previously^63^ as well as including iTRAQ peptide labeling as a fixed modification. Identified proteins were further validated and quantitated using Scaffold Q^+^ software, version 4.0.4 (Proteome Software, Portland, OR). For protein identification, a minimum of two unique peptides, a MOWSE score ≥32, peptide probability ≥90% and protein threshold ≥95% were considered. Proteins were compared between each iTRAQ data set and considered for comparative analysis if a protein was identified in the two data sets. Changes in protein abundance were calculated as fold change from average value obtained from all replicates of each sample. A change in abundance was then determined in comparison with the corresponding controls (white-fluorescent −114 and −116 iTRAQ tags). To increase confidence level for functional analysis, only proteins that have *P* ≤ 0.05 (Student’s T-test) and a fold change of Ι1.5Ι in all three technical replicates and are consistent across the two biological replicates were considered as significant and subjected to gene ontology (GO) analysis.

### Chemicals and statistical analysis

All chemicals were purchased from Sigma-Aldrich, St. Louis, MO, unless stated otherwise. Statistical analysis was performed using Student’s *t*-test with Microsoft Excel 2010. Significance was set to a threshold of *P* < 0.05 and *n* values represent the number of biological replicates.

## Additional Information

**How to cite this article**: Ooi, A. *et al*. Growth and development of *Arabidopsis thaliana* under single-wavelength red and blue laser light. *Sci. Rep.* **6**, 33885; doi: 10.1038/srep33885 (2016).

## Supplementary Material

Supplementary Information

## Figures and Tables

**Table 1:** Primers and PCR conditions.

Primer Name	Sequence (5′–3′)	Tm (°C)	No. of cycle
*psbA* qPCR forward	TGCCATTATTCCTACTTCTGCA	60	30
*psbA* qPCR reverse	AGCACTAAAAAGGGAGCCG	60	30
*psaA* qPCR forward	GCAGGGCTACTAGGACTTGG	60	30
*psaA* qPCR reverse	GGCCTGTAAATGGACCTTTATG	60	30
*petA* qPCR forward	CAGCAGAATTATGAAAATCCACG	60	30
*petA* qPCR reverse	TATTAGTAGCAGGGTCTGGAGCA	60	30
*ATFD2* qPCR forward	ACTTCATTCATCCGTCGTTCC	60	30
*ATFD2* qPCR reverse	AAGAACCAGCACGGCAAG	60	30
*LHCB1.1* qPCR forward	CCGTGTGACAATGAGGAAGA	60	30
*LHCB1.1* qPCR reverse	CAAACTGCCTCTCCAAACTTG	60	30
*ATBCA3* qPCR forward	CGAGTTCATAGAAAACTGGATCC	56	35
*ATBCA3* qPCR reverse	AGGCAGGGGTAGTCTTGAAGT	56	35
*APX1* qPCR forward	GGACGATGCCACAAGGATA	58	35
*APX1* qPCR reverse	GTATTTCTCGACCAAAGGACG	58	35
*GST* qPCR forward	TCTATAAAACACCATACCTTCCTTCA	58	35
*GST* qPCR reverse	CGAAAAGCGTCAAATCACC	58	35
*PP2AAC* qPCR forward	GCGGTTGTGGAGAACATGATACG	*	*
*PP2AAC* qPCR reverse	GAACCAAACACAATTCGTTGCTG	*	*

^*^The annealing temperature and PCR cycles of *PP2AAC* ‘housekeeping’ gene is dependent on the PCR condition of the genes being studied.
