# Supplementary material for: Growth and development of Arabidopsis thaliana under single-wavelength red and blue laser light
Source: Sci Rep. 2016 Sep 23;6:33885. doi: 10.1038/srep33885 (PMC5034235; doi:10.1038/srep33885)

Supplementary Information

Growth and development of *Arabidopsis thaliana* under single-wavelength red and blue laser light

Authors

Amanda Ooi[1]*, Aloysius Wong[1]*, Tien Khee Ng[2], Claudius Marondedze[3], Christoph Gehring[1] & Boon Ooi[2]†

Affiliations

[1] Division of Biological and Environmental Sciences and Engineering, 4700 King Abdullah University of Science and Technology, Thuwal 23955-6900, Kingdom of Saudi Arabia.

[2] Division of Computer, Electrical, and Mathematical Sciences and Engineering, 4700 King Abdullah University of Science and Technology, Thuwal 23955-6900, Kingdom of Saudi Arabia.

[3] Department of Biochemistry, Cambridge Centre for Proteomics, University of Cambridge, Cambridge, United Kingdom.

†To whom correspondence should be addressed: boon.ooi@kaust.edu.sa

* These authors contributed equally to this work.

Supplementary Figure S1

Seedling emergence of *Arabidopsis thaliana* under different regime of LEDs for optimization of spectral composition.

(**a**) Physical measurements of (**i**) hypocotyl length and (**ii**) percentage of seed germination and (**b**) biochemical analysis of (**i**) total chlorophyll and (**ii**) total carotenoid level of 5-days old Col-0 seedlings to determine the optimal spectral composition, i.e. ratio and light components, for the growth and development of *Arabidopsis thaliana*. Sterile Col-0 seeds were stratified at 4°C in the dark prior sowing to half-strength Murashige and Skoog (MS) agar medium (with sucrose, pH 5.7). Plants were germinated and grown under different LED light compositions and intensities for a period of five days in a continuous light regime or for 16/8 hour photoperiod at 22°C with a relative humidity of 50-60 %. Hypocotyl length was measured using imageJ software. Error bars represent standard error of the mean calculated from n > 18, where n represents independent biological replicates. (**c**) 5-days old *Arabidopsis* seedlings grown under cool-white fluorescent (W) and red and blue (RB) LED light regime (9:1 ratio of red (670 nm peak) and blue (470 nm peak)) respectively at an average photon flux density of 22 µmol m^{-2} s^{-1} for 16/8-hour photoperiod.

Supplementary Figure S2

Phenotypic and biochemical characterisation of plants grown under a 9:1 ratio of red and blue LED light.

(**a**) Phenotypic observation of (**i**) 14-days old and (**ii**) 21-days old *Arabidopsis* plants grown under the LED light regime (9:1 ratio of red (670 nm peak) and blue (470 nm peak) LED (RB) light) at an average photon flux density of 75 µmol m^{-2} s^{-1} for 16/8-hour photoperiod at 22 °C with a relative humidity of 50–60 %. RB LED-grown plants displayed distinct phenotype, i.e. elongated petiole length, broader and expanded leaf blade as compared to the control plants that were grown under white fluorescent (W) light. (**b**) Measurement of (**i**) fresh and (**ii**) dry weight and (**c**) biochemical analysis of (**i**) total chlorophyll and (**ii**) carotenoid level of 22-days old RB LED-grown plants. Error bars represent standard error of the mean calculated from 10 independent biological replicates.

Supplementary Figure S3

Spectral composition and chromaticity diagram of cool-white fluorescent and red and blue laser light.

The spectral composition of (**a**) the cool-white fluorescent (W), and (**b**) red and blue DPSS lasers (RB) used in this study. Irradiance spectra of each respective light source were measured *in situ* using a GL SPECTICS 5.0 Touch spectrometer (JUST Normlicht GmbH, Weilheim an der Teck, Germany) within the waveband range from 340 to 850 nm. The graphs were plotted using a OriginPro 8.5.1 SR2 software (OriginLab Corporation, Northampton, MA). The corresponding chromaticity diagrams for (W) and (RB) light are shown in (**c**), and (**d**), respectively. The (W) light has CIE (*Commission Internationale de l'Eclairage*) 1931 color coordinates (black cross), correlated color

temperature, and color rendering index of (0.39,0.38), 3879, and 82.4 respectively. These values are used to quantify human perception of light quality. As for the (RB) light, the dosage is more important than the perceived color of light to confirm the narrow spectrum of the laser characteristic, as indicated by the color coordinates (0.50, 0.47).

Supplementary Figure S4

Seedling emergence and root development of plants grown under red and blue laser and LEDs.

(**a**) 14-days old *Arabidopsis* plants that were germinated and grown on half-strength MS agar medium (without sucrose, pH 5.7) under (**i**) cool-white fluorescent, (**ii**) red and blue (RB) laser light regime (9:1 ratio of red (671 nm) and blue (473 nm) lasers) and (**iii**) red and blue (RB) LED light regime (9:1 ratio of red (670 nm peak) and blue (470 nm peak)) at an average photon flux density of 80–90 µmol m^{-2} s^{-1} for 16/8-hour photoperiod at 22 °C with a relative humidity of 50–60 %. (**b**) Root development of 14-days old *Arabidopsis* plants under different light regime. Root length was measured using a 15 cm ruler. Error bars represent standard error of the mean calculated from three independent experiments (n = 30) where n represents independent biological replicates for each experiment.

Supplementary Figure S5

Growth and development of laser-treated F1 offspring.

(**a**) 7-days old and (**b**) 14-days old laser-treated *Arabidopsis* F1 offspring that were germinated and grown on half-strength MS agar medium (with sucrose, pH 5.7) under the red and blue (RB) laser light regime (9:1 ratio of red (671 nm) and blue (473 nm) lasers) at an average photon flux density of 90–100 µmol m^{-2} s^{-1} for 16/8-hour photoperiod at 22 °C with a relative humidity of 50–60 %. (**c**) 21-days old and (**d**) 35-days old of laser-treated F1 offspring transferred from (**b**) to soil-based growth, in which these plants were further subjected to growth under cool-white fluorescent (W) light at similar light intensity and growth condition upon transfer from the (RB) laser light regime.

Supplementary Figure S1

Seedling emergence of *Arabidopsis thaliana* under different regime of LEDs for optimization of spectral composition.

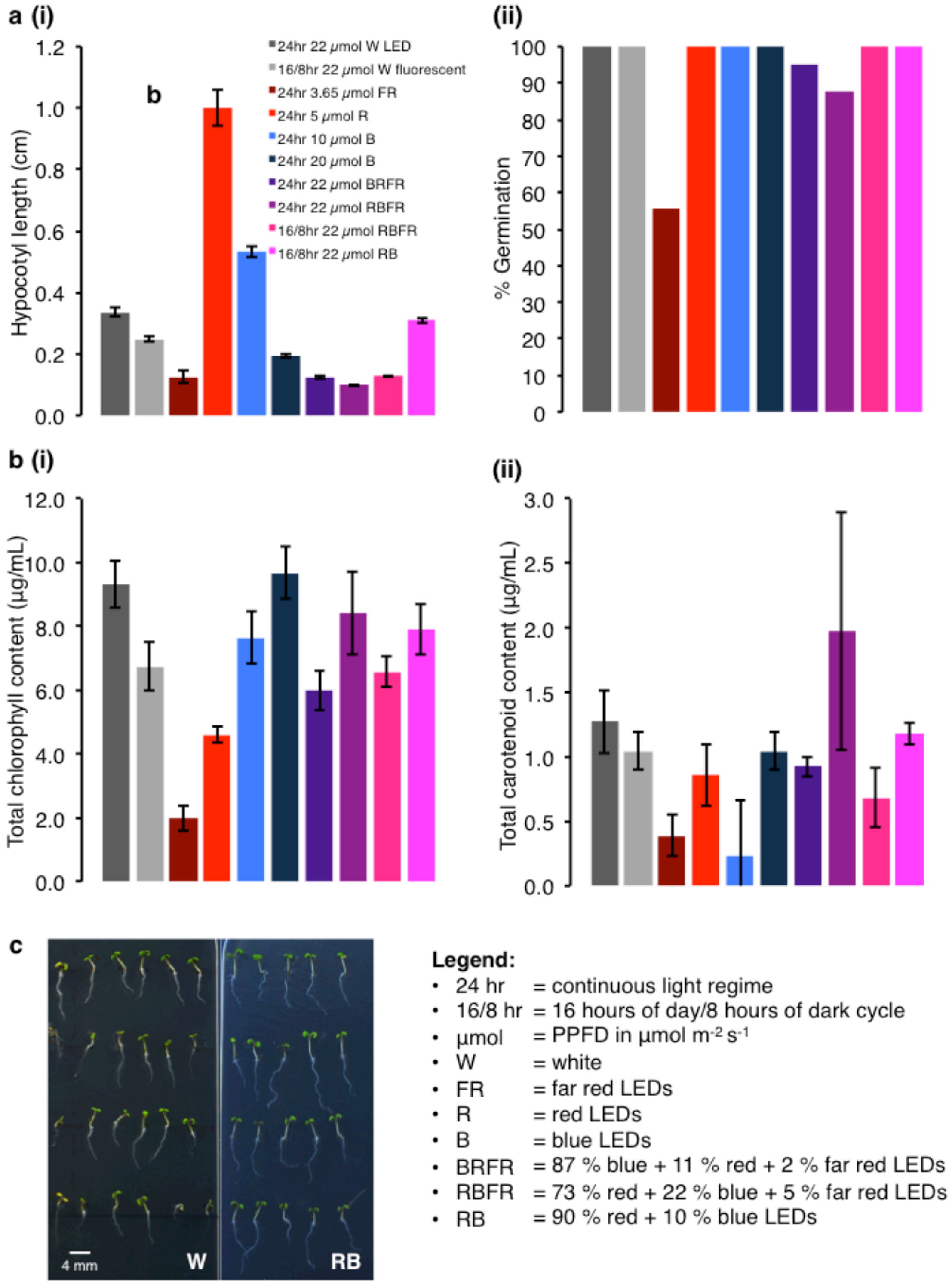

Supplementary Figure S2

Phenotypic and biochemical characterisation of plants grown under a 9:1 ratio of red and blue LED light.

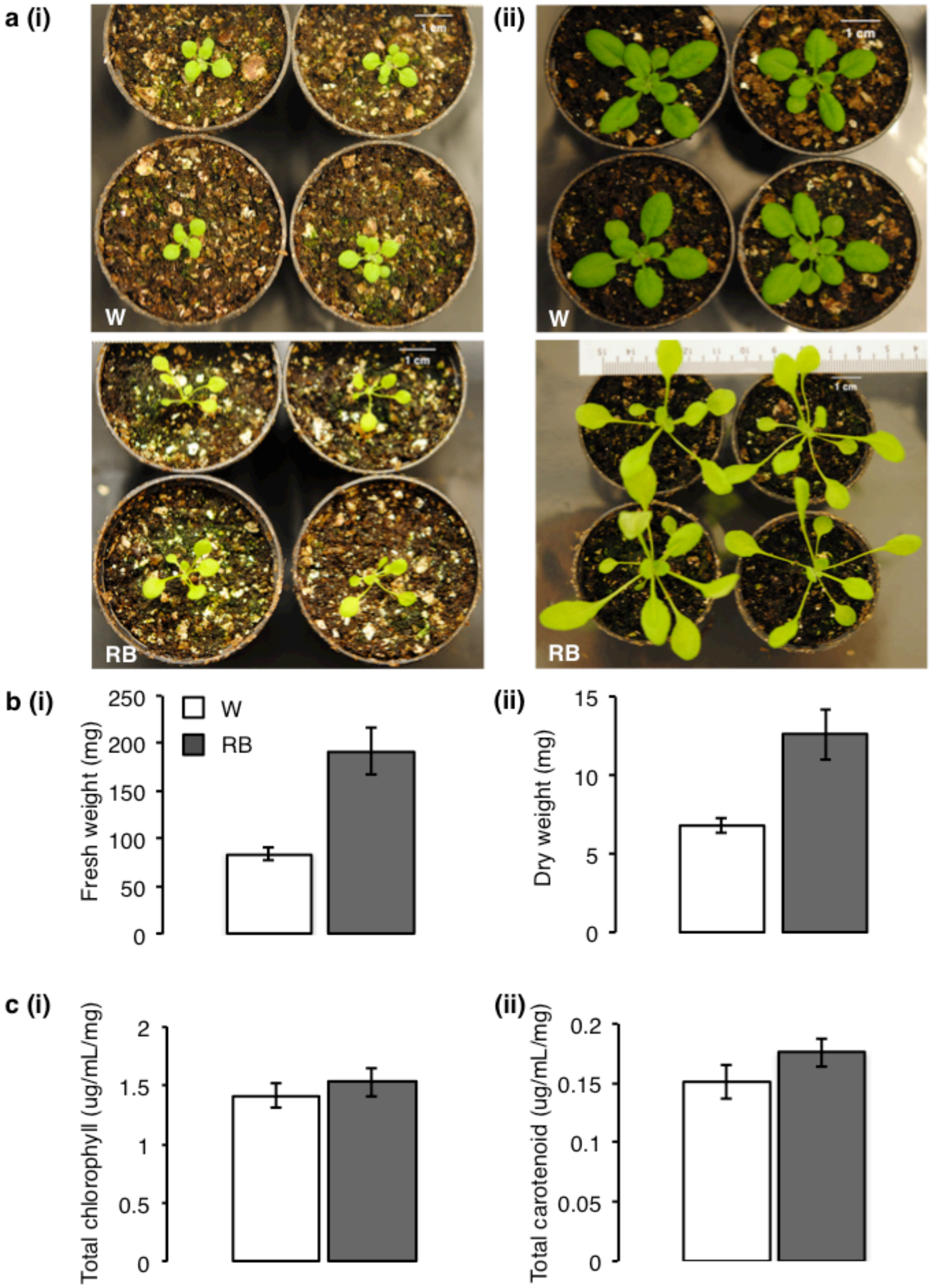

Supplementary Figure S3

Spectral composition and chromaticity diagram of cool-white fluorescent and red and blue laser light.

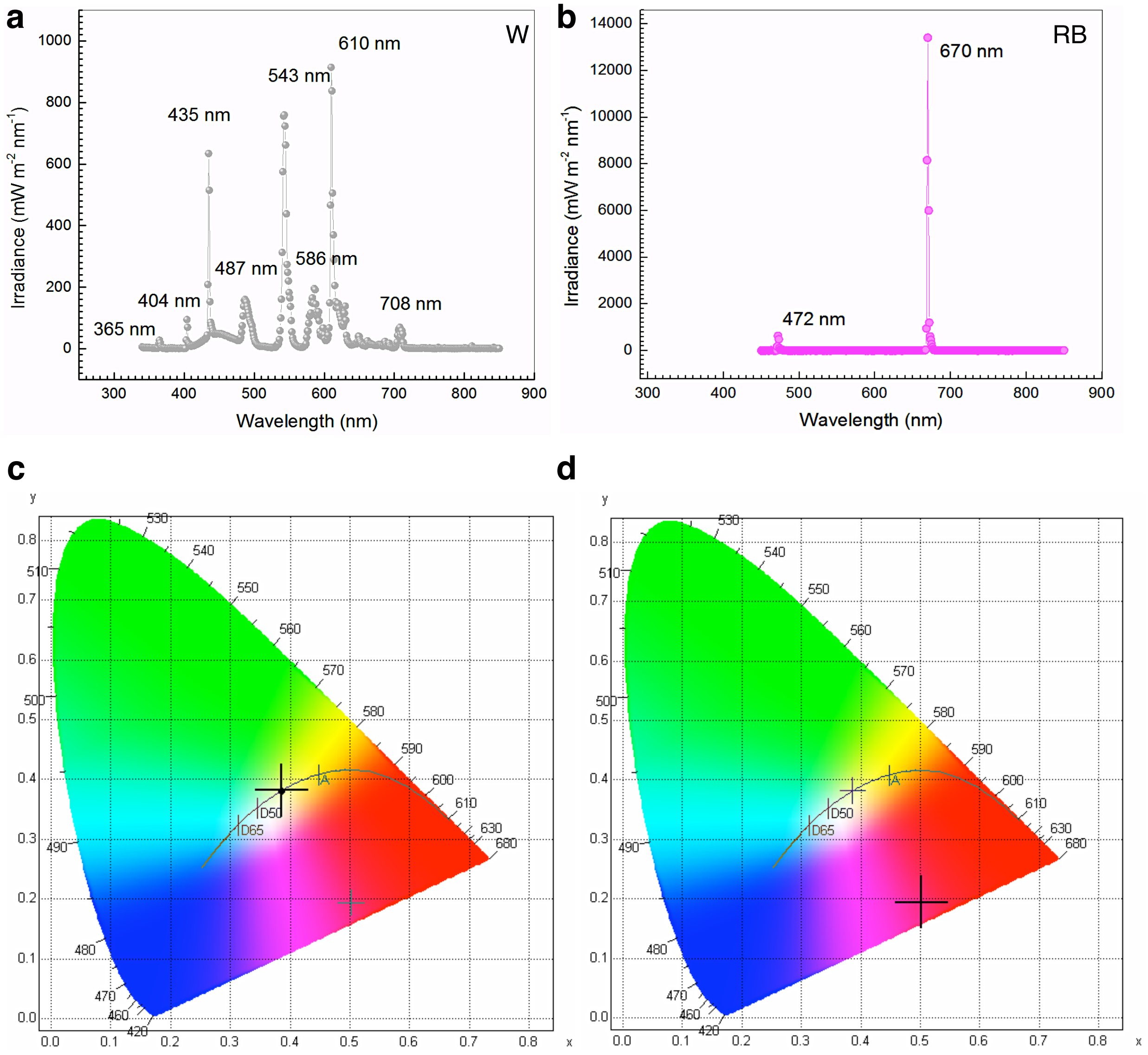

Supplementary Figure S4

Seedling emergence and root development of plants grown under red and blue laser and LEDs.

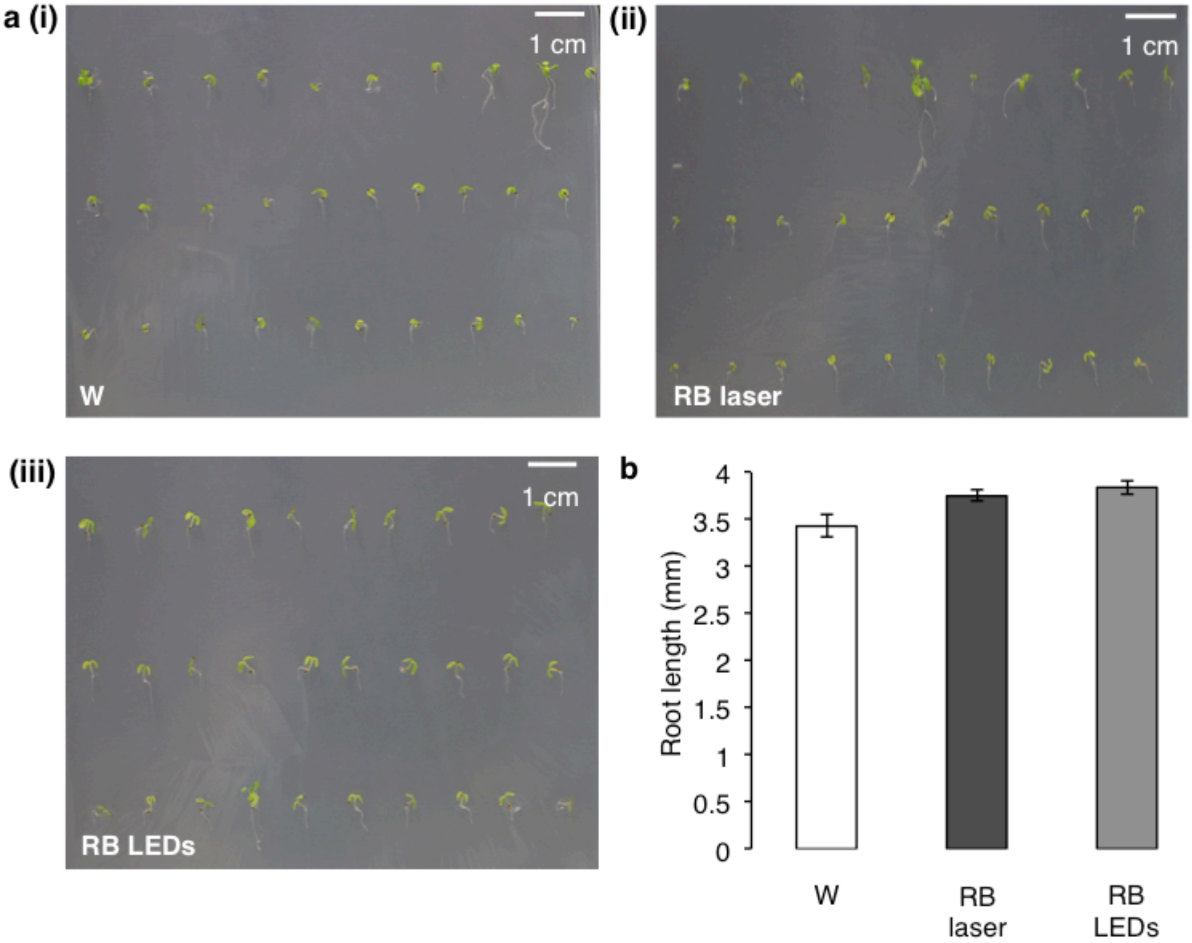

Supplementary Figure S5

Growth and development of laser-treated F1 offspring.

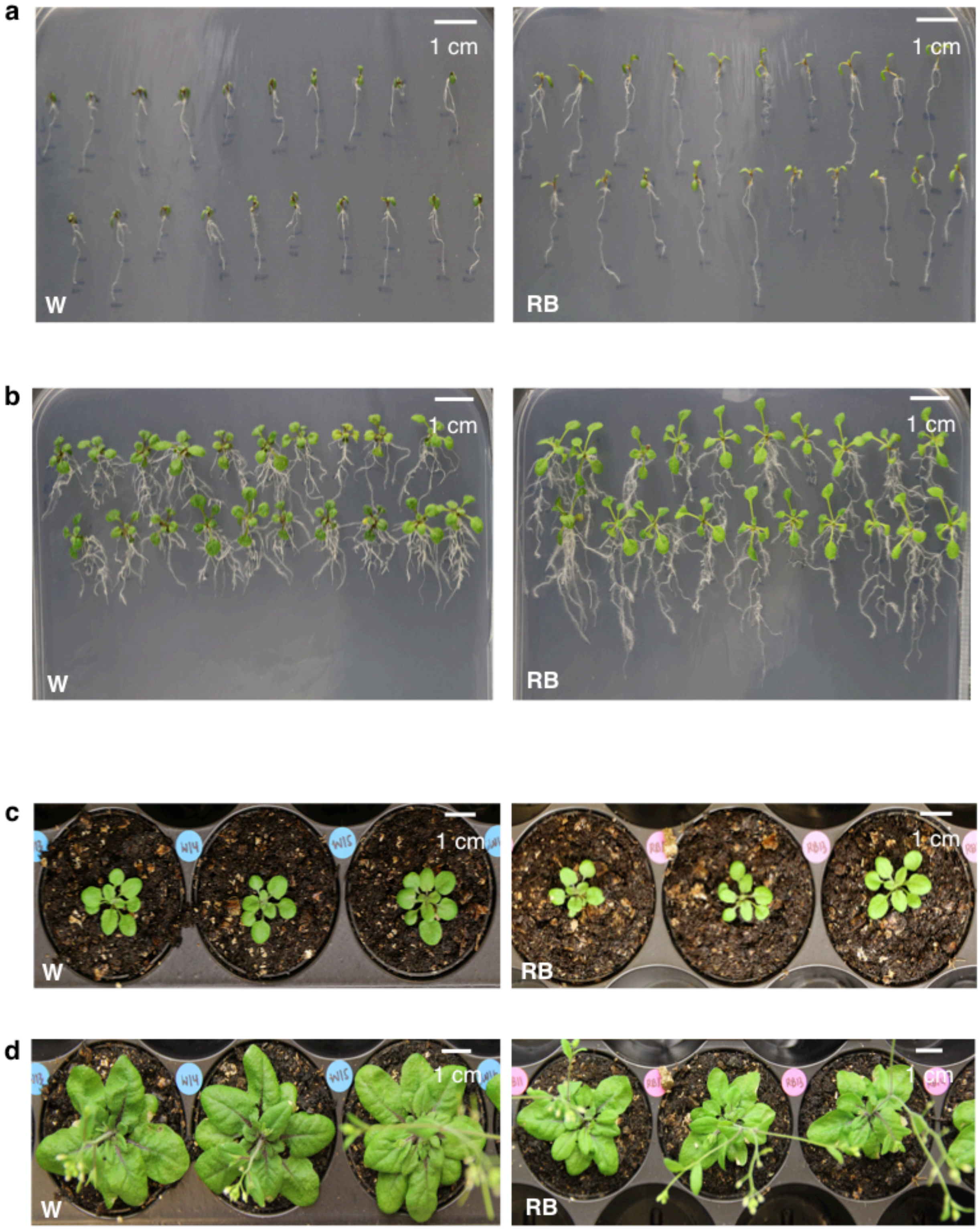

Supplement: Supplementary Information [file srep33885-s1.pdf]
